# Achieving Targeted Delivery of Chemotherapeutic Particles to Small Airway Tumors via Pulmonary Route Using Endotracheal Catheters: A CFPD Study

**DOI:** 10.3390/ph16020158

**Published:** 2023-01-22

**Authors:** Mohammad Rashedul Islam, Yu Feng

**Affiliations:** School of Chemical Engineering, Oklahoma State University, Stillwater, OK 74078, USA

**Keywords:** computational fluid particle dynamics (CFPD), targeted pulmonary drug delivery, lung cancer, endotracheal catheter

## Abstract

Tracheobronchial tumors, while uncommon, are often malignant in adults. Surgical removal is the primary therapy for non-metastatic lung malignancies, but it is only possible in a small percentage of non-small-cell lung cancer patients and is limited by the number and location of tumors, as well as the patient’s overall health. This study proposes an alternative treatment: administering aerosolized chemotherapeutic particles via the pulmonary route using endotracheal catheters to target lung tumors. To improve delivery efficiency to the lesion, it is essential to understand local drug deposition and particle transport dynamics. This study uses an experimentally validated computational fluid particle dynamics (CFPD) model to simulate the transport and deposition of inhaled chemotherapeutic particles in a 3-dimensional tracheobronchial tree with 10 generations (G). Based on the particle release maps, targeted drug delivery strategies are proposed to enhance particle deposition at two lung tumor sites in G10. Results indicate that controlled drug release can improve particle delivery efficiencies at both targeted regions. The use of endotracheal catheters significantly affects particle delivery efficiencies in targeted tumors. The parametric analysis shows that using smaller catheters can deliver more than 74% of particles to targeted tumor sites, depending on the location of the tumor and the catheter diameter used, compared to less than 1% using conventional particle administration methods. Furthermore, the results indicate that particle release time has a significant impact on particle deposition under the same inhalation profile. This study serves as a first step in understanding the impact of catheter diameter on localized endotracheal injection for targeting tumors in small lung airways.

## 1. Introduction

Lung cancer is one of the deadliest illnesses today. According to the American Cancer Society, lung cancer is responsible for 25% of all cancer-related deaths in the United States, more than colon, breast, and prostate cancers combined [1]. While tumors in the tracheobronchial (TB) tree are rare, accounting for less than 0.4% of all body cancers, [2,3], still 90% of these tumors are malignant in adults and 30% are malignant in children [4]. Surgery and radiation therapy are commonly used to remove these tumors, but they can be invasive and may not be an option for some patients [5]. Chemotherapy is another method typically needed for killing cancer cells, maintaining after-treatment health, and extending longevity for whom the treatments mentioned above are not viable options [6,7]. Among multiple types of chemotherapy, a novel chemotherapeutic approach, that is, delivery of chemotherapeutic microparticles via the pulmonary route, has been shown to be effective against lung cancer [8,9]. In 1983, a study found that the vaporized anti-cancer drug 5-Fluorouracil (5-FU) had a good response in treating cancer when inhaled into the lung tree [9]. Similar results were found in a separate study on dogs [8]. Both studies showed that the retention of inhaled isotope tracers was longer in tumor tissue than in healthy tissue, which helps to explain the medication’s strong anti-tumor effects and the lack of side effects from inhaled 5-FU. Huland et al. [10] found in a study that interleukin-2 (IL-2) therapy for cancer patients may be most effective when administered locally by inhalation, resulting in a high quality of life for patients as lung metastases did not advance. Otterson et al. [11] studied 53 patients with malignant lung illness who were administered 13 doses of inhalable doxorubicin, ranging from 0.4 to 9.4 mg/m^2^. According to their findings, individuals with cancer who had normal to moderately reduced pulmonary function were safe using inhaled doxorubicin up to a dosage of 7.5 mg/m^2^ every three weeks. Researchers also compared how much cancer treatment drugs accumulated by intravenous injection and inhalation, while developing an inhalation co-delivery of anti-cancer drugs and siRNA [12]. The animal study found that inhalation delivery retained 73% of siRNA-loaded nanoparticles in the lungs, while intravenous injection retained 73% in the liver, indicating that inhalation delivery may result in greater accumulation of the drug in tumors [12]. According to a different study, inhalable nanoparticle drugs containing doxorubicin improved the survival period of the tumor-bearing mice model compared to the other group that received the same dose of medicine via intravenous administration [13]. Overall, administering chemotherapeutic particles through the pulmonary route can be an effective treatment for lung cancer.

Inhalation chemotherapy can still be potentially harmful, as a large amount of particles can deposit on healthy tissues, leading to severe side effects. [14]. Therefore, it is necessary to achieve targeted delivery, that is, increase the deposition of chemotherapeutic particles on designated tumor sites, and reduce the deposition on healthy tissues. Regional targeting via inhalation is a method of providing treatments into the lungs, with increased drug particle deposition in specific lung sites, typically those with the affected or diseased areas, including cancer cells [15]. When designing effective inhalable drugs, particle characteristics, including particle size, density, and surface composition, are crucial since these help to determine the location of the deposits. However, achieving targeted delivery of chemotherapeutic particles to small-airway tumors via oral inhalation is challenging. One drawback is the difficulty in avoiding drug particle deposition in the upper airway, due to the curvature from the mouth to the larynx and the presence of the glottis, which induce strong inertial impaction and turbulence dispersion [16]. Additionally, the lung deposition of most commercially available dry powder inhalers (DPIs) ranges from 10–40%, with a significant portion remaining in the mouth-to-throat region [17,18].

To avoid deposition in the upper airway and healthy tissues with no therapeutic effect, an alternative method is to use an endotracheal catheter injection, which involves directly releasing the medication from the trachea into the small airways [19]. Endotracheal catheters are typically made of medical grade materials such as polyvinyl chloride (PVC), silicone, or Teflon. PVC is commonly used in disposable catheters due to its low cost and easy to sterilize. Silicone is a popular choice for reusable catheters due to its biocompatibility and flexibility. Teflon is less common, but it is known for its durability and resistance to kinking. Additionally, some endotracheal catheter may have a coating or surface modification to prevent bacterial adherence. The choice of materials used for the endotracheal catheter depends on the desired properties, such as flexibility, durability, and biocompatibility. Using endotracheal catheters to release particles at locations downstream to the trachea can avoid upper airway deposition due to the inertial impaction and laminar-to-turbulence transitional flow dispersion, and can potentially deliver more particles to small airways. Researchers explored catheters in the urinary route [20] and the central venous route [21] to treat cancers. Non-surgical catheter-based treatments, including radiofrequency ablation (RFA), microwave ablation (MWA), cryoablation (CRA), and photodynamic therapy (PDT), are being developed as possible solutions [22]. However, the feasibility of using an endotracheal catheter to achieve targeted delivery of chemotherapeutic particles to small-airway tumors has not been systematically studied.

To determine the feasibility of targeted delivery using endotracheal catheters for treating small-airway tumors, it is important to understand how chemotherapeutic particles transport and deposit in lung airways interacting with pulmonary airflow. Obtaining high-resolution, quantitative details of these dynamics through in vitro or in vivo studies can be difficult due to limitations in imaging resolution, operational flexibility, and ethical considerations. As an alternative, computational fluid particle dynamics (CFPD) can be used to predict air-particle flow dynamics in lung airways. This approach has been researched for decades to improve our understanding of the complex mechanisms involved in respiratory drug delivery [23]. As a physics-based computational model, experimentally validated CFPD can significantly reduce the time and expense required to develop in vivo and in vitro models, while evaluating targeted drug delivery systems. It can precisely determine particle trajectories for various inhalation conditions. Research efforts have been made using CFPD to achieve pulmonary targeted drug delivery, such as the Controlled Particle Release and Targeting (CPRT) approach introduced by Kleinstreuer and Zhang [24]. This approach is used to develop new inhalation devices for the most efficient aerosolized medication delivery to a designated lung site in the human respiratory system. Specifically, by using the “backtracking” strategy [24], the precise particles release coordinates were predetermined to deliver the drug particles to the targeted region. The research was able to confirm a significant increase in particle deposition fractions (DFs) for the targeted region in lung airways can be achieved using the CPRT method compared with the conventional particle injection method. Another in silico study used a similar CPRT concept to achieve lobe-specific drug delivery and showed that it is possible to improve drug DF in any specific lobe by up to 90% when the releasing position and velocity of the drug particles were fine-tuned [14]. Additionally, drug delivery via the pulmonary route was also combined with magnetic substances to achieve targeted delivery and improve aerosol deposition utilizing magnetic fields that were delivered externally [25,26,27,28]. Another CFPD study introduced a novel inhalation therapy of a short-pulsed bolus of aerosolized drug particles [29]. The study claimed that this drug delivery method can lead to drug delivery efficiency to small airways (i.e., G7 and above) higher than 68%.

In addition to the above-mentioned CFPD research efforts on achieving pulmonary targeted drug delivery, there have been only a few other CFPD studies that have examined particle trajectories and airflow patterns in airways with lung tumors. For example, one study used a CFPD model to construct bifurcations with lung carcinoma tumors on carinas and investigated the effects of tumor size and ventilatory characteristics on regional flow behavior to assist targeted delivery of inhaled medications [30]. Another CFD-based study suggested that an efficient targeted drug delivery system would benefit from understanding the impact of aerosol deposition on the location of the tumor [31]. However, there have been no CFPD studies on targeted drug delivery treatments for small-airway tumors using endotracheal catheters. Since the feasibility of the pulmonary delivery method using endotracheal catheter for small-airway tumor targeting has not yet been well-studied, it is crucial to understand how to modulate the particle release parameters to achieve targeted delivery to tumors in small airways. It is also important to investigate which parameters can significantly influence drug delivery efficiency to the tumor sites.

**Figure 1:**
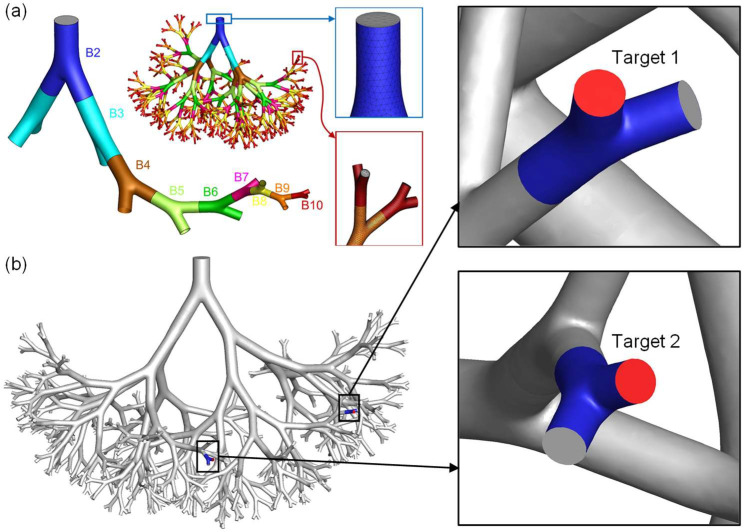
Full 3D airway tree geometry and mesh details: (**a**) Schematic of the different levels of bifurcations and the mesh details, (**b**) positions of the two randomly selected tumor locations.

Therefore, using a targeted delivery strategy similar to the CPRT method [24] which is controlled injection using endotracheal catheters, the objective of this study is to investigate how particle release time, ventilation flow rate, and catheter diameter and position can influence the targeted delivery efficiency (DE) to the tumors in small airways, using an experimentally validated one-way coupled Euler–Lagrange-based CFPD model [32]. Specifically, an idealized 3D airway geometry was employed in this study, which covers 10 generations (Gs) from bifurcation 2 (B2) to bifurcation (B10), which represents the anatomical features of young adult male patients who may have a higher possibility of having benign small-airway tumors than adults (see Figure 1). To investigate the general effectiveness of the proposed targeted drug delivery for distant tumors at different locations, two tumor sites (i.e., Target 1 and Target 2) were selected at B10 randomly at different locations far from each other. Particle release maps were generated to determine the catheter release position for targeted drug delivery based on conventional injection simulation results. Two catheters with different inner diameters of 0.1 mm and 2.5 mm were selected compared with the conventional injection in DE to the targeted tumor sites. The conventional injection has the same opening of the B2 inlet, which is 8.5 mm. To obtain insightful and statistically robust conclusions on the objectives, this study also investigated how different particle release times and inhalation flow rates’ effects targeted drug delivery using catheter injection. Transient sinusoidal inhalation–exhalation waveforms with different average flow rates were applied (see Section 3.3). This study is innovative as it is the first to employ a CFPD approach to evaluate the feasibility of using controlled particle injection with endotracheal catheters for targeting small-airway tumors. It fills a significant gap in the understanding of targeted drug delivery for small-airway tumors by providing novel insights on how different parameters, such as particle release time, ventilation flow rate, and catheter diameter and position affect targeted DE. The findings of this study may aid in the development of more effective pulmonary targeted drug delivery systems for treating cancerous tumors in the human respiratory system.

## 2. Results and Discussion

### 2.1. Conventional Injection vs. Controlled Particle Injection Using Endotracheal Catheters

**Figure 2:**
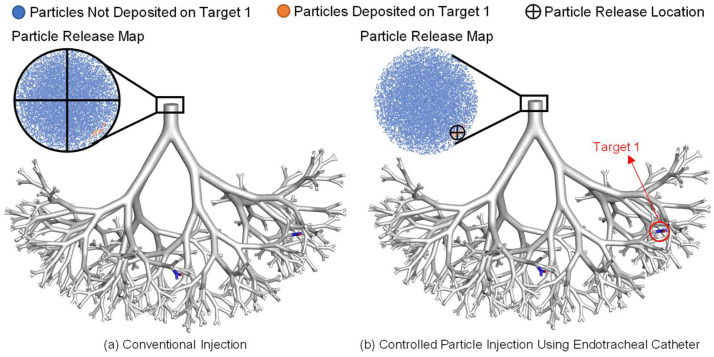
Demonstration of the difference in conventional injection vs. catheter injection (catheter position is determined based on the particle release map generated using conventional injection method): (**a**) Conventional injection, and (**b**) controlled particle injection using endotracheal catheters.

Using Target 1 as an example, Figure 2 illustrates the difference and relationship between the conventional injection method and the controlled injection method using endotracheal catheters. The particle release maps were first generated using the “back-tracking” strategy based on conventional injection simulations. Specifically, for the conventional injection simulations, particles with aerodynamic diameters of 1 µm were randomly positioned and injected at the inlet. The conventional injection method utilizes the same inlet diameter as the geometric model used in this study and backtracks the initial particle injection position to generate the release map (as shown in Figure 2a). The particle release maps, generated based on the conventional injection simulation results, visualize the initial injection locations of particles at the inlets, colored differently based on their deposition locations. For example, the orange region shown in Figure 2a represents the initial position of those particles injected that were deposited on Target 1. Non-targeted particles are colored blue. The particle release map generated was used to determine the catheter position for the controlled particle injection method (as shown in Figure 2b). Specifically, to achieve targeted delivery to Target 1, the catheter opening should be located at a position with the largest overlap to the orange region, which is the optimal location for particle release to target the specific tumor site.

### 2.2. Particle Release Maps vs. Average Inhalation Flow Rate and Particle Release Time

To investigate the effect of the average inhalation flow rate and particle release time on targeted drug delivery efficiency (DE) to small-airway tumors, conventional injection simulations were conducted. Three average inhalation flow rates (30 L/min, 45 L/min, and 60 L/min) and three particle release times (t = 0.0 s, 0.5 s, and 1.0 s) were simulated using 1 µm particles with aerodynamic diameters between 0.5 and 2 µm, which have been found to have the best potential for transport to small airways [32,33]. Specifically, three average inhalation flow rates were simulated with the transient sinusoidal inhalation–exhalation waveform (see Section 3.3). The results were used to generate particle release maps and DEs for two randomly selected tumor locations (Target 1 and Target 2) to determine the optimal conditions for targeted delivery using controlled particle injection with endotracheal catheters. More details can be found in Section 3.2 of the paper.

**Figure 3:**
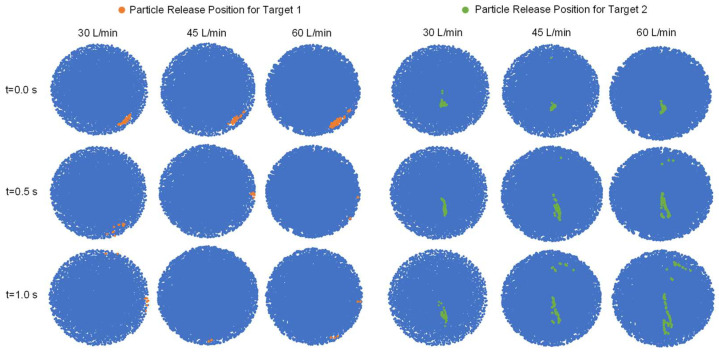
Particle release maps generated using conventional injection simulations with varied average inhalation flow rates (30, 45, and 60 L/min) and particle release times (0.0, 0.5, and 1.0 s).

Figure 3 illustrates the impact of average inhalation flow rate and particle release time on the available regions for particle release positions targeting the two tumors (i.e., orange regions for Target 1 and green regions for Target 2) in the particle release maps. It can be observed that as the particle release time increases from 0.0 s to 1.0 s, the available regions for particle release positions to target both tumors become more scattered. Notably, the available regions are more concentrated and larger in area when the particle release time is set at t = 0.0 s, providing more flexibility for releasing therapeutic particles for targeted delivery. Therefore, for controlled particle injection using endotracheal catheters, a t = 0.0 s particle release time was chosen in this study. The particle release maps also show that the targeting area changed very slightly when compared for different average inhalation flow rates ranging from 30 to 60 L/min with a particle release time of 0.0 s. In contrast, with the same particle release times t = 0.5 s and t = 1.0 s, increasing the average inhalation flow rate induced more scattered available regions for particle release positions to target both tumors. Indeed, increasing the average inhalation flow rate and particle release time before reaching the inhalation peak flow rate in the sinusoidal waveform (see Section 3.3) will increase the turbulence dispersion effects and lead to stronger particle trajectory deviations from following the laminarized streamlines, which is the reason for the more scattered available regions for particle release positions to target both tumors. The findings reconfirm the fact that the laminar flow regime is more ideal for the realization of controlled particle injection for targeted drug delivery via the pulmonary route [14].

### 2.3. Targeted Particle DE vs. Average Inhalation Flow Rate and Particle Release Time

**Figure 4:**
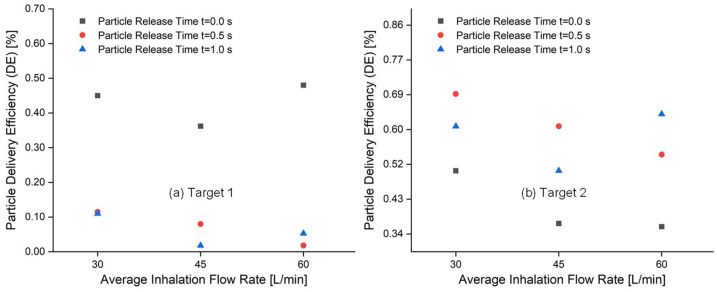
Comparison of targeted particle delivery efficiencies (DEs) for Target 1 and Target 2 using the conventional injection method at varying flow rates and particle release times: (**a**) Target 1, and (**b**) Target 2.

To further examine the effects of average inhalation flow rate and particle release time on targeted particle DE for both tumors, Figure 4 compares the targeted particle DEs for Target 1 and Target 2 based on the simulation results obtained from the conventional injection method. It can be seen from Figure 4a,b that both average inhalation flow rate and particle release time have an impact on the particle DEs for both Target 1 and Target 2. However, the effects are not consistent between the two targets and the influence of these parameters on Target 1 and Target 2 differ.

It can be observed that increasing average inhalation flow rates decrease the particle DEs on both Target 1 and Target 2, as shown in Figure 4. However, there are two exceptions for both tumors. As seen in Figure 4a, at a particle release time of t = 0.0 s and t = 1.0 s, an increase in average inhalation flow rate from 45 L/min to 60 L/min improves the particle DE on Target 1. Conversely, Figure 4b shows that at a particle release time of t = 1.0 s, an increase in average inhalation flow rate from 45 L/min to 60 L/min enhances the particle DE on Target 2. The particle release time also has a significant impact on the DEs for all average inhalation flow rates. For Target 1, the highest particle DEs can be achieved when particles are released at t = 0.0 s for all average inhalation flow rates, as seen in Figure 4a. An inconsistency is observed in Figure 4b where it is shown that the lowest particle DEs are achieved when the particle release time is set to t = 0.0 s for all average inhalation flow rates. Instead, the highest DE on Target 2 is associated with particle release time t = 0.5 s at 30 L/min. Clearly, the average inhalation flow rate and particle release time has an influence on particle deposition. However, the DE changes for Target 1 and Target 2 related to these two parameters are not consistent and are dependent on the particle release time at different flow rates. Further study is required to find a better correlation in between particle release time and the particle DEs in small-airway tumors as a function of tumor locations, which is related to the different catheter release position and the local airflow velocity magnitude. For Target 1 drug delivery, the available region for controlled particle injection is near the wall of the B2 inlet. Comparatively, the available region for particle injection to aim for Target 2 is near the center of the B2 inlet. Due to the stronger viscous effect near the airway wall, higher average inhalation flow rates may not induce as strong of a turbulence dispersion effect for particles injected to be delivered to Target 1 as compared to Target 2.

Furthermore, it can be observed in Figure 4 that the highest DE achieved using conventional injection method is 0.48% for Target 1 and 0.69% for Target 2. This suggests that conventional injection alone is not an efficient method for delivering chemotherapeutic particles to small-airway tumors and can result in high particle depositions on healthy tissue, leading to severe side effects. Therefore, using controlled particle injection with an endotracheal catheter is necessary, as shown in Figure 2b. The targeted delivery strategy must be tailored to the specific location of the tumor.

To determine the optimal average inhalation flow rate and particle release time for targeted delivery to both tumors using controlled particle injection with an endotracheal catheter, the two parameters that resulted in the highest particle DEs were used as one of the criteria. As shown in Figure 4a, Target 1 achieved the highest particle DE at 60 L/min and particle release time t = 0.0 s. Similarly, Figure 4b shows that Target 2 achieved the highest particle DE at 30 L/min and particle release time t = 0.5 s. Additionally, consideration was given to selecting the largest available regions for particle release positions to target both tumors (as seen in Figure 3). Therefore, 60 L/min and t = 0.0 s were chosen to deliver particles to Target 1, while 30 L/min and t = 0.0 s were selected for Target 2. The corresponding particle release maps were then used to determine the positions of catheters with different diameters for targeted drug delivery.

### 2.4. Targeted Particle DE vs. Catheter Diameter

**Figure 5:**
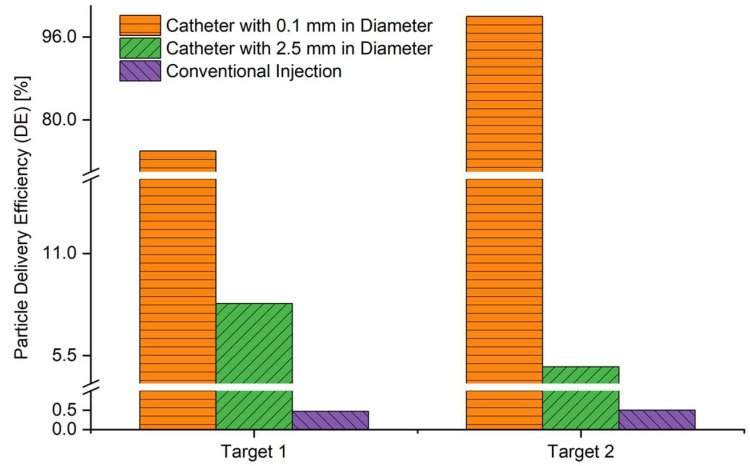
Comparison of targeted particle DEs on both tumors between conventional injection method and controlled particle injection methods using catheters with different diameters.

After the determination of average flow rate and particle release time for optimal particle delivery to both tumors, catheter diameter influences on the effectiveness of the controlled particle injection method were further studied. Specifically, catheters with two different diameters (i.e., 2.5 mm and 0.1 mm) were employed, and targeted particle DEs on both tumors were compared with the conventional injection (see Figure 5). Coordinates of the catheter center were determined using the available regions for particle release positions shown in Figure 3. More details can be found in Section 2.5. DE comparisons shown in Figure 5 clearly show that controlled particle injection using endotracheal catheters can significantly enhance the particle DEs on tumors compared with the conventional injection method. Catheters 0.1 mm in diameter can deliver much more particles to both targets compared with catheters 2.5 mm in diameter. Specifically, catheters 0.1 mm in diameter deliver 100% of particles to Target 2 with 30 L/min and around 74% particles on Target 1 with 60 L/min. In contrast, catheters 2.5 mm in diameter lead to 8.3% and 4.9% DE on Target 1 and Target 2, respectively. As mentioned in previous sections, only 0.47% and 0.5% of the particles can be delivered to Target 1 and Target 2 using the conventional injection method, respectively. These findings suggest that reducing the catheter diameter can potentially increase the targeted delivery efficiency to small-airway tumors, with more precise control of the particle trajectories. It is also worth mentioning that although 0.1 mm catheters show the best targeted delivery performance, the manufacture of such small endotracheal catheters is challenging. To the best of our knowledge, the smallest catheter diameter that is commonly used in clinical practice is 2.5 mm. Therefore, to achieve better targeted delivery efficiency using inhalation chemotherapy, the capability of manufacturing catheters with smaller openings (e.g., 0.1 mm in diameter) needs to be improved. To create an endotracheal catheter with a diameter less than 0.1 mm, silicone could be the best material due to its biocompatibility and flexibility. Silicone has a high level of elasticity and can be formed into very thin and small shapes while maintaining its structural integrity. It also has a low coefficient of friction which is beneficial for the insertion and maneuvering of small catheters. However, it is worth noting that it would be challenging to manufacture such small catheters with high precision and accuracy, and the catheter might not be strong enough to withstand the insertion, maneuvering and handling during the procedure. Furthermore, it would be hard to sterilize such small catheters and the risk of bacterial contamination might increase.

The low particle delivery efficiency on both tumors using conventional injection methods can be attributed to the particle release maps as shown in Figure 2 and Figure 3. As shown in Figure 2a, conventional injection uses the entire B2 inlet opening for particle injection, resulting in most particles being injected from the blue region in the particle release maps. These particles are unlikely to reach the designated tumor sites. On the other hand, when particles are injected using catheters (see Figure 2b), the radius of the injection area is much smaller, allowing for particles to be injected from the orange or green regions shown in Figure 3, increasing the likelihood of particles reaching the designated tumor sites. The dynamics of particle transport are discussed in more detail in Section 2.5.

### 2.5. Particle Transport Dynamics with Controlled Injection Using Endotracheal Catheters

**Figure 6:**
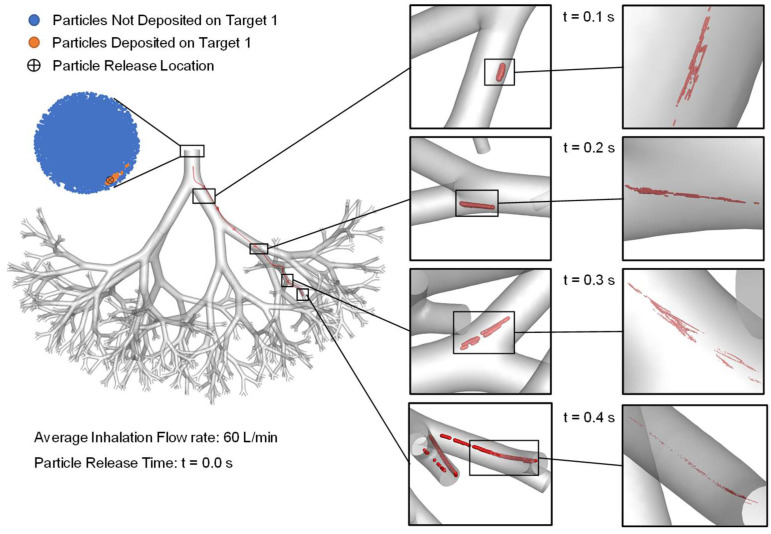
Particle transport dynamics for the targeted delivery simulations using the controlled particle injection method with 0.1 mm endotracheal catheter to aim for Target 1.

**Figure 7:**
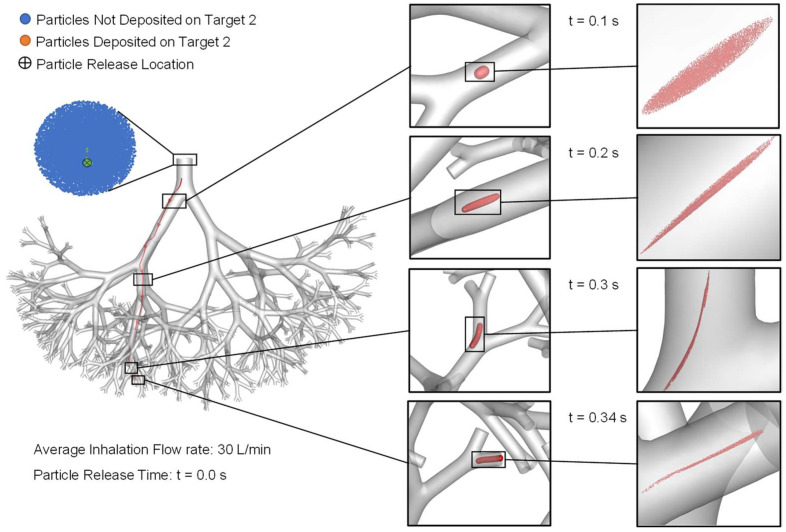
Particle transport dynamics for the targeted delivery simulations using the controlled particle injection method with a 0.1 mm endotracheal catheter to aim Target 2.

Although using small catheters can significantly increase the particle DE on tumors, it is worth mentioning that the DE on Target 1 with a catheter 0.1 mm in diameter is 74% instead of 100%, as was achieved for Target 2. To further study the reason for such a difference in DE, particle transport dynamics for simulations using 0.1 mm catheters for Target 1 and Target 2 are visualized in Figure 6 and Figure 7, respectively. It is worth mentioning that although the simulation case shown in Figure 6 has a higher inlet average inhalation flow rate (i.e., 60 L/min) than Figure 7 (i.e., 30 L/min), the particle delivery time to Target 1 and Target 2 are similar, that is, approximately 0.4 s. This is due to the similar local airflow velocity magnitudes at the two catheter positions shown in Figure 6 and Figure 7. Specifically, although the average inhalation flow rate for catheter injection to aim for Target 1 (see Figure 6) is 2 times the simulation case to aim for Target 2 (see Figure 7), the catheter position of the simulation shown in Figure 6 is much closer to the wall than the simulation shown in Figure 7. The stronger viscous dissipation leads to a relatively lower velocity magnitude of the near-wall airflow and the particles during their transport.

Furthermore, it can also be observed from Figure 6 that injected particles initially follow the streamline well until t = 0.3 s, heading towards the B10 connected with Target 1. The particle cloud became more and more stretched due to the shear flow effect induced by local airflow velocity gradients close to the airway boundaries. Eventually, the particle cloud bifurcated into both daughter tubes in the B10 connected to Target 1 at t = 0.4 s. Combining with the DE shown in Figure 5, 26% of the particles bifurcated into the airway in that B10 and did not deposit on Target 1. In comparison, although particle cloud shown in Figure 7 also stretched due the shear flow effect, 100% of the particle transport through the daughter tube connected with Target 2.

By examining the particle release maps shown in Figure 3 more closely, it can be observed that the available region for particle injection to aim for Target 2 (i.e., the green regions in Figure 3) is highly concentrated with particles. Accordingly, placing the 0.1 mm catheter anywhere in the green region results in 100% particle deposition. However, the particle concentration in the available region for particle injection to aim for Target 1 (i.e., the orange regions in Figure 3) has a blank inner space, which can be the potential regions from where injecting particles may head to other destinations in small airways than Target 1, as shown in the particle locations at t = 0.4 s in Figure 6. Injecting more particles at the B2 inlet for generating the particle release maps using a conventional injection method may be able to provide more details on the topology of the orange region for optimization of the catheter position to further enhance the targeted particle delivery efficiency to Target 1.

Based on the results and analysis in this section, it can be concluded that the average inhalation flow rate, particle release time, catheter diameter, and tumor locations can all significantly influence targeted particle DEs and delivery strategy optimizations. Controlling the particle injection locations with the preferred local airflow velocity field is key to achieving highly targeted delivery efficiency to designated tumor sites.

## 3. Materials and Methods

### 3.1. Geometry and Mesh

Figure 1 shows an idealized three-dimensional (3D) 10-generation airway geometry of a portion of the TB tree. For this study, the airway inlet was scaled to the same hydraulic diameter as G2, which is equal to 8.5 mm of a young adult male subject [34]. The diameter at G10 is 1.348 mm. For targeted delivery simulations, transient sinusoidal inhalation–exhalation waveforms were employed. The average inhalation flow rates range from 30 to 60 L/min.

Ansys Fluent Meshing 2021 R1 (Ansys Inc., Canonsburg, PA, USA) was used to create unstructured tetrahedral-based CFD meshes. To resolve the airflow velocity boundary layers, five near-wall prism layers were generated and refined. Four meshes were created for the grid independence test, and the specifications of all meshes are listed in Table 1. The final mesh contains 10,641,093 cells, 25,530,968 faces, and 4,428,041 nodes. For the mesh independence test, the inlet airflow Reynolds number (Re_in_) was set to 4055 corresponding to an inhalation rate Q_in_ = 120 L/min at the mouth as an extreme condition. The mouth inlet velocity was 7.044 m/s correspondingly. Zero-gauge pressures were applied at all outlets. No-slip velocity boundary conditions were applied at all airway walls. The mesh independence test was performed using steady-state airflow simulation. The details of the final tetrahedral mesh selected for this study are shown in Figure 1a.

**Figure 8:**
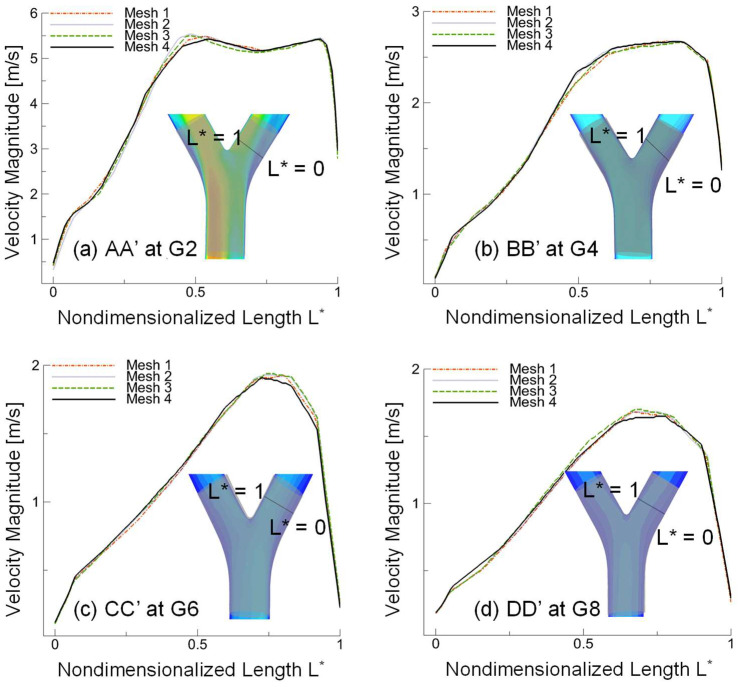
Velocity profiles at four different generations, (**a**) AA’ cross-section at G2, (**b**) BB’ cross-section at G4, (**c**) CC’ cross-section at G6, and (**d**) DD’ cross-section at G8.

To determine the mesh independence, multiple lines were taken at four individual planes at G2, G4, G6, and G8, which are shown in Figure 8a–d. The comparisons of velocity profiles shown in Figure 8a–d reveal that there are no significant differences in velocity predictions between Mesh 2 and Mesh 3 with further refinement. Therefore, Mesh 2 was accepted as the final mesh for this study, based on the optimal balance between computational accuracy and efficiency.

### 3.2. Governing Equations

Pulmonary airflow is assumed to be incompressible with constant viscosity and density. The pressure-based coupled solver was used to simulate the ambient airflow field using a set of equations comprising conservation rules. The governing equations of the two-phase flow are given in tensor form, that is,

(1) $$\frac{\partial u_{i}}{\partial x_{i}}=0$$

(2) $$\frac{\partial u_{i}}{\partial t}+u_{j}\frac{\partial u_{i}}{\partial x_{j}}=-\frac{1}{\rho }\frac{\partial p}{\partial x_{i}}+\frac{1}{\rho }\frac{\partial \tau_{ij}}{\partial x_{j}}+g_{i}$$

in which *ρ* is the density of air, *p* is the pressure, *u_i_* denotes the air velocity, and *g_i_* is the gravitational acceleration vector. Specifically, gravity is directed in the positive x-direction. The viscous stress tensor *τ_ij_* is defined by:

(3) $$\tau_{ij}=\mu \left(\frac{\partial u_{i}}{\partial x_{j}}+\frac{\partial u_{j}}{\partial x_{i}}\right)$$

where *μ* is the air viscosity.

To predict particle trajectories, a Lagrangian approach is employed in this simulation, which is also known as the discrete phase model (DPM) [35]. A one-way coupled Euler–Lagrange model is employed in this study, indicating that the flow field influences the fluid movement, but the flow field is not affected by the presence of the particles. A total of 11,330 particles were injected at the B2 inlet. Particles of 1 μm were chosen for this study, as particle aerodynamic diameters ranging from 0.5–2 μm are usually deposited in small airways [33]. The particles were considered spherical with a density of 993 kg/m^3^. The rotational motion was ignored due to the small particle size. The particle translation equation for each particle was solved to obtain their trajectories, that is,

(4) $$\frac{d}{dt}\left(m_{p}{\vec{u}}_{p}\right)={\vec{F}}_{D}+{\vec{F}}_{BM}+{\vec{F}}_{G}$$

where $m_{p}\;$ and $\;{\vec{u}}_{p}$ are the mass and velocity of the particle, respectively; $\;{\vec{F}}_{G}$ and $\;{\vec{F}}_{D}$ are the gravity and drag force, respectively. Brownian motion induced force ${\vec{F}}_{BM}$ is negligible for particles with 1 μm in aerodynamic diameter. The drag force $\;{\vec{F}}_{D}$ can be given by:

(5) $${\vec{F}}_{D}=\frac{1}{8}\pi \rho d_{p}^{2}C_{D}{\left(\vec{u}-{\vec{u}}_{p}\right)}^{2}/C_{c}$$

in which $d_{p}$ is the particle diameter, $C_{c}$ is the Cunningham correction factor, and $C_{D}\;$ is the drag coefficient, which is defined as [36]:

(6) $$C_{D}=a_{1}+\frac{a_{2}}{Re_{p}}+\frac{a_{3}}{Re_{p}}$$

where $a_{1}$, $a_{2}$, and $a_{3}$ are the constants and can be determined by a particle Reynold’s number. Morsi et al. [37] defined $Re_{p}$ as the particle Reynold’s number which can be expressed as:

(7) $$Re_{p}=\frac{\rho \left|{\vec{u}}_{p-}\vec{u}\right|}{\mu }$$

The Cunningham correction factor $C_{c}$ can be given by [38]:

(8) $$C_{c}=1+\frac{2\lambda }{d_{\;p}}\left(1.257+0.4e^{-1.1\frac{d_{p}}{2\lambda }}\right)$$

in which λ is the mean free path of air.

### 3.3. Initial and Boundary Conditions

**Figure 9:**
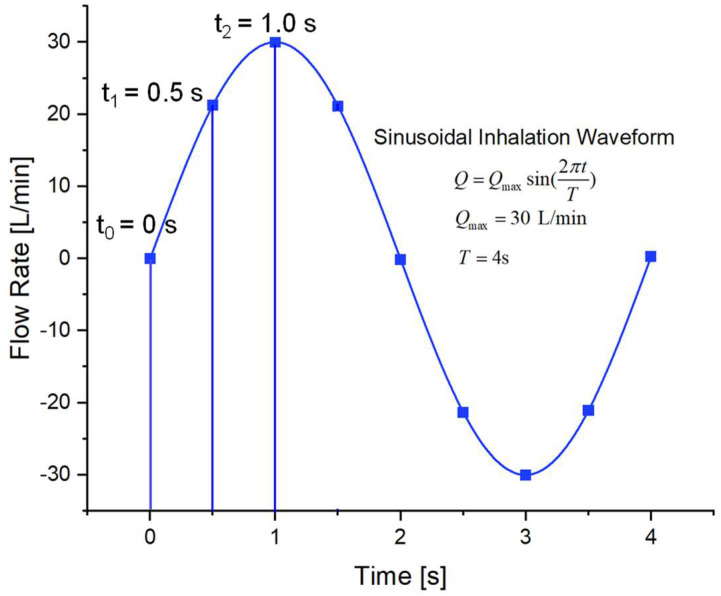
Sinusoidal inhalation–exhalation waveform.

An idealized sinusoidal velocity profile with an inhalation–exhalation ratio of 1:1 was employed at the inlet position G2 [39]. All five lobes of the lung were assumed to get an equal distribution of the air flow rates. As a result, the inlet air flow at G2 was equal to 1/5 and the inhaled air flow rate varied from 1.76 to 3.52 m/s for three different flow rates considered in this study. Specifically, the sinusoidal waveform of the inhalation–exhalation flow rate Q is defined as (see Figure 9):

(9) $$Q=Q_{max}\;\sin\left(\frac{2\pi t}{T}\right)$$

where $Q_{max}$ is the amplitude of the waveform indicating the highest flow rate, and T is the total inhalation–exhalation time which is considered 4 s in this model.

In the CFPD simulation, zero-gauge pressures were applied at all outlets and the walls of the airway were assumed to be static. The non-slip velocity boundary condition was applied to the airway walls. For particle deposition, an “escape” boundary condition was applied at both the inlet and outlets, while the lung airway walls were considered 100% trapped due to the presence of mucus.

### 3.4. Particle Targeted Delivery Efficiency (DE)

To evaluate the effectiveness of the targeted drug delivery strategy, the targeted particle delivery efficiency (DE) of the chemotherapeutic particles was used as a metric, which is defined as

(10) $$DE=\frac{Number\;of\;particles\;delivered\;to\;a\;specific\;region}{Total\;number\;of\;particles\;injected\;at\;the\;inlet}$$

### 3.5. Numerical Setup

The multilevel bifurcation lung airway aerosol transport model was conducted using Ansys Fluent 2021 R1 (Ansys Inc., Canonsburg, PA, USA). The finite-volume method and second-order discretization techniques were employed to solve the equations both in space and time. To ensure numerical stability, a flow time-step of 0.001 s was used and the simulation was considered to have converged once all residuals fell below 1.0 × 10^−4^. The numerical simulations were performed on a Dell Precision T7810 workstation (featuring Intel^®^ Xeon^®^ Processor E5-2643 v4 with dual processors, 64 cores and 128 GB RAM) and a Dell Precision 7920 workstation (equipped with Intel^®^ Xeon^®^ Silver 4116 with dual processors, 64 cores, and 256 GB RAM).

In-house user-enhanced programs were developed and used to customize the solver, and achieved the following functions, that is:

- Defining the transient sinusoidal inhalation waveforms (see Figure 9);
- Defining the DPM time-step;
- Defining the DPM drag coefficient; and
- Output of post-processing particle deposition data in the lung airway.

### 3.6. Model Validations

It is worth noting that the one-way coupled Euler–Lagrange model used in this study and the in-house codes have been thoroughly validated in previous publications [32,40,41,42]. These validations include comparisons with benchmark experimental data, such as:

1. The Transition Shear-Stress Transport (SST) model employed in this study to predict laminar-to-turbulence flow regime has been validated in a previous study [43]. Specifically, comparisons of airflow velocity magnitude and iso-surfaces show good matches between the employed SST k-ω transition model, and the experimental measurements [44] were performed in the same subject-specific human airway model.
2. The CFPD model used in the current study was also employed to predict the discrete-phase transport and deposition, as can be seen in Figure S2 in the supplemental material of a previously published work [45]. The predicted regional deposition efficiencies (RDEs) were then compared to benchmark the experimental data obtained from physical models of lung airways, with inhalation flow rates ranging from 15 to 60 L/min. The comparisons revealed a high level of agreement between the results obtained from the numerical and experimental studies.

## 4. Conclusions

This study used an experimentally validated CFPD model to investigate the feasibility of targeted chemotherapeutic particle delivery using an endotracheal catheter to target tumors located in small airways (G10). The study also quantified the impact of factors such as average inhalation flow rate, particle release time, and catheter diameter on the particle release map and particle delivery efficiency to tumors. CFPD was used to compare the deposition of 1 µm particles in nine bifurcations of a human airway model. The developed model was used to analyze the DF in three different inhalation flow rates at three different particle release times to target two different tumor regions. The targeted delivery was analyzed using two types of catheter injection and compared to conventional particle release methods. By optimizing the particle administration parameters based on the particle release maps generated from conventional injection simulations inside a full 3D G2–G10 airway tree geometry, the targeted particle DEs on tumors were significantly enhanced. Key conclusions are listed below.

1. The conventional injection method can only deliver less than 1% of the chemotherapeutic particles to the designated tumor sites, which indicates a strong side effect will be caused due to the high percentage of particle depositions on healthy tissues from G2 to G10. In contrast, a controlled particle injection method was proposed using a 0.1 mm endotracheal catheter, which can improve the particle DEs on Target 1 and Target 2 to 74% and 100%, respectively.
2. Both average inhalation flow rate and particle release time had an influence on the topology of particle release maps. With the increase in local airflow velocities surrounding the particle release (i.e., increase in average inhalation flow rate or increase the particle release time during the sinusoidal inhalation phase before reaching the peak flow rate), the available regions for particle release for achieved targeted delivery to both tumors became more scattered, due to the enhanced turbulence dispersion effect.
3. Both average inhalation flow rates and particle release time had an impact on the particle DEs on both tumors. However, the influences were different between Target 1 and Target 2, which were highly dependent on tumor locations. Therefore, the targeted delivery strategy is dependent on tumor locations.
4. With the decrease in catheter diameter, the targeted particle DEs on both tumors increased.
5. For seeking the optimal average inhalation flow rate and particle release time for the controlled particle injection method using endotracheal catheters, two criteria were used, that is, (1) highest particle DEs, and (2) largest areas of available regions for particle release position to aim for the tumor. For Target 1, 60 L/min and a 0.0 s particle release time were employed. For Target 2, 30 L/min and a 0.0 s particle release time were selected.

## 5. Limitations of this Study and Future Work

The current numerical study employed certain simplifications and assumptions, such as:

1. The small lung airway model used in the study is stationary and does not take into account any lung movement;
2. The study did not consider the effects of cilia-driven mucus motion on medication clearance; and
3. The dynamics of medication perfusion into tumor cells following deposition were not modeled in the study.

To address the limitations of the current study, future research will incorporate the movement of airways using a dynamic mesh method and fluid–structure interactions (FSI) with an experimentally validated elastic whole-lung modeling framework [46,47]. This will enable the capture of physiologically realistic kinematics of healthy and diseased airways and its impact on targeted drug delivery efficiency. Additionally, the effect of mucus clearance on drug delivery will be investigated using a multiscale numerical method that integrates CFD, the Discrete Element Method (DEM), and Volume of Fluid (VOF) [48,49]. Furthermore, the perfusion of medication into tumors will be modeled using a method similar to CFPD, plus the Physiologically-Based Pharmacokinetic (PBPK) model.

## Figures and Tables

**Table 1:** Details of the meshes used for the independence test.

Mesh No.	Nodes	Faces	Cells	Near-Wall Prism Layers
1	2,817,233	15,052,480	6,059,303	5
2 (Final)	4,428,041	25,530,968	10,641,093	5
3	4,962,751	29,349,686	12,357,045	5
4	5,493,502	33,328,366	14,168,216	5

## Data Availability

The data that support the findings of this study are available from the corresponding author upon reasonable request.

## Abbreviations

The following abbreviations are used in this manuscript:

5-FU	5-Fluorouracil
B	Bifurcation
CFD	Computational Fluid Dynamics
CFPD	Computational Fluid Particle Dynamics
COVID-19	Coronavirus Disease 2019
CRA	Cryoablation
CPRT	Controlled Particle Release and Targeting
DF	Deposition Fraction
DE	Delivery Efficiency
DPI	Dry Powder Inhaler
DPM	Discrete Phase Model
FSI	Fluid-Structure Interaction
G	Generation
IL-2	Interleukin-2
MWA	Microwave Ablation
PDT	Photodynamic Therapy
PVC	Polyvinyl Chloride
RDE	Regional Deposition Efficiency
RFA	Route Radiofrequency Ablation
SST	Shear Stress Transport
TB	Tracheobronchial
VOF	Volume of Fluid
